# Optimising Electrode Montages in Conventional Transcranial Direct Current Stimulation and High-Definition Transcranial Direct Current Stimulation of the Cerebellum for Pain Modulation

**DOI:** 10.3390/brainsci15040344

**Published:** 2025-03-27

**Authors:** Adelais Farnell Sharp, Alice Witney

**Affiliations:** 1 Trinity College Institute of Neuroscience (TCIN), Trinity College Dublin, D02 R590 Dublin, Ireland; afarnell@tcd.ie; 2 Academic Unit of Neurology (AUoN), School of Medicine, Faculty of Health Sciences, Trinity College Dublin, D02 R590 Dublin, Ireland

**Keywords:** cerebellum, neurostimulation, TDCS, HD-TDCS, pain, nociception, current-modelling, targeting, simulation, lobes, anode

## Abstract

The cerebellum is involved in pain processing and, therefore, an important target for non-invasive brain stimulation (NIBS) for analgesia. When targeting a brain region for NIBS, it can be difficult to ensure activation of only target regions. Optimal Montages for cerebellar stimulation for pain modulation have not been established. This paper systematically examines cerebellar NIBS Montages by comparing simulated current flow models for targeted conventional cerebellar tDCS and focused high-definition 4 × 1 HD-tDCS, to examine the most effective Montage for targeting only the lobes of the cerebellum. The objective was to explore if slight variations in electrode placement and voltage could be producing confounding activations of other brain regions as shown by the Soterix^®^ current modelling software (Ver. 2019). A left deltoid anode for right cerebellar lobe sponge (3 cm lateral to inion) produces the best targeting with conventional tDCS. For high-definition tDCS (HD-tDCS) a 4 × 1 array based on a 93-electrode EEG map, with the central electrode at PO10, and the array at O2, P8, Ex2, and Ex6, provided focal stimulation. Optimisation of NIBS must include an evaluation of electrode Montages and current flow modelling to determine which structures and pathways will be impacted by the neurostimulation. This approach is essential for future cerebellar NIBS experimental design and will facilitate comparative analysis across different protocols and optimise understanding of the role of the cerebellum in pain processing.

## 1. Introduction

Pain is a complex, multi-faceted experience, with multiple brain regions implicated in its processing. The IASP defines pain as “An unpleasant sensory and emotional experience associated with, or resembling that associated with, actual or potential tissue damage” [1,2]. Pain is considered a multi-factorial phenomenon with independent physical, emotional, and cognitive components [3]. A proposed purpose of pain is to help an organism detect threats to its survival and adjust behaviour to prevent further injury [1].

### 1.1. Activation of the Cerebellum During Pain Stimulation in Humans

For an organism to withdraw from painful stimuli, there needs to be integration of nociceptive and motor processing. This is a postulated role for the cerebellum in the pain matrix. The cerebellum receives ascending information from the spinal cord and descending responses from other brain regions (including the thalamus, somatosensory cortex, and motor cortex) putting it in an integrative position with access to comprehensive sensory information [4]. Numerous fMRI studies have shown activation of the cerebellum following application of noxious stimuli of varying modalities and it is considered one of the most reliable areas of the brain in terms of reacting to painful stimuli [4,5].

The cerebellar cortex is organised into two lobes which receive projections from multiple brain regions. Evidence suggests that it is somatotopically organised. Wellman et al. [6] used fMRI to identify regions of activation following painful stimulation of the right toe or right thumb and compared to activations that occurred in anticipation of pain in the same areas. They found overlapping activation for anticipatory and actual pain for each area (thumb anticipation and thumb stimulation overlapped in Lobule VI of the cerebellum) suggesting specialised areas are primed for specific inputs from localised parts of the body.

One theory for the cerebellums role in nociceptive processing is that it is primed by pain to prepare a motor response such as withdrawal. Coombs et al. [7] used fMRI to chart activated regions following noxious stimuli and found significant clusters of activity across both motor and cognitive sub-regions of the cerebellar cortex. They concluded that it was consistent with the notion that pain primed for adaptive behavioural response, showing the possible integration of stimuli and environmental information.

Another theory is that it attempts to distinguish types of noxious stimuli. Helmchen et al. [8] used fMRI to study activation of the cerebellum in response to both noxious and innocuous thermal stimuli and determine whether it processed actively applied and passively applied pain differently. They found activation was modulated in line with stimulus intensity in actively applied conditions but not passively. This suggests the cerebellum has distinguished between the two types of stimuli independent of motor response generation, suggesting higher order processing.

Baumann et al. [4] postulated that it acts as an integration centre for nociceptive and motor information, allowing for the generation of a withdrawal reflex response. FMRI studies have found significant clusters of activity across both motor and cognitive sub-regions of the cerebellar cortex following noxious stimuli [8], consistent with the notion that pain primes for adaptive behavioural response such as withdrawal. Moulton et al. [3] argued that the cerebellum is specifically related to generation of a motor response to pain. They hypothesised that cerebellar responses to noxious stimuli in neuroimaging 17 studies could be ascribed to inhibition of the escape response as people must stay still during scans. They used fMRI to look at cerebellar responses to noxious heat stimulation of the hand and the viewing of emotionally unpleasant images. They found overlap of activation across conditions and believe this indicates that the cerebellum is involved in encoding generally aversive stimuli and preparing to react, rather than higher processing.

Voluntary inhibition cannot fully explain findings as noxious heat can induce cerebellar activation even under anaesthesia. Hofbauer et al. [9] took PET scans of the cerebellum after administration of varying doses of propofol, an anaesthetic agent that alters pain perception. They found significant cerebellar activation regardless of sedation level, and that the activation continued to be significant even after unconsciousness. These findings suggest that the cerebellar response to pain is involuntary and does not require conscious perception.

An alternative explanation for integration of stimulus in the cerebellum is that it is the result of an attempt to distinguish types of noxious stimuli. Helmchen et al. [8] used fMRI to study activation of the cerebellum in response to noxious and innocuous thermal stimuli and determine whether it processed actively applied and passively applied pain differently. They found activation was modulated in line with stimulus intensity in actively applied conditions but not passively. This suggests the cerebellum has distinguished between the two types of stimuli independent of motor response generation, suggesting higher order processing. Finally, it appears that specialised areas of the cerebellum may be primed for specific inputs. Welman et al. [6] used fMRI to identify regions of activation following painful stimulation of the right toe or right thumb and compared to activations that occurred in anticipation of pain in the same areas. They found overlapping activation for anticipatory and actual pain for each area (thumb anticipation and thumb stimulation overlapped in Lobule VI) suggesting each area concerned itself with a localised part of the body.

**Figure 1:**
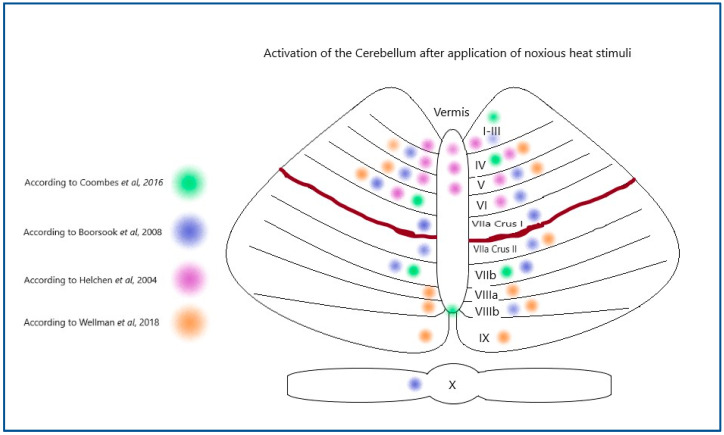
Areas of activation in response to noxious stimuli noted on fMRI studies mapped onto the anatomy of the cerebellum from a dorsal view. Original created for this paper [5,6,7,10].

When you compare the regions of activation throughout the research literature (see Figure 1), the cerebellum is consistently activated, but the activation is not limited to a single area.

### 1.2. Neurostimulation Targeting in the Cerebellum

For the cerebellum to be a valid target it would need to repeatedly demonstrate reliable activation following pain stimuli. Borsook et al. [5] claims the cerebellum “always” demonstrates activation in human neuroimaging studies of pain and may be involved in integrative processing differentiating noxious form innocuous stimuli. Welman et al. [6], Coombes et al. [7], and Helmchen et al. [10] all recorded activation of the cerebellum using fMRI following application of noxious thermal stimuli, supporting the cerebellum as a viable target.

Although the cerebellum does, therefore, present as a potentially viable target, it is important to note that as well as there being a lack of clarity on the cerebellum’s role and contribution to pain perception, there is also a gap in research investigating how ctDCS specifically influences the cerebellum on a functional level. The depth of current penetration for example, could affect where noted effects of ctDCS of the cerebellum are generated (whether from the cerebellar cortex, deep nuclei, or white matter) [11] and could influence stimulator placement and contribute to knowledge of which specific areas of the cerebellar cortex are the best targets [11].

However, as promising as results appear, limitations of the cerebellum as a target must be considered. Often motor activation recorded via EMG is used as indication of successful excitation of M1. Patients with cerebellar lesions that affect movement often show little interference with nociception, and there is no known clinical condition of altered pain processing due to cerebellar lesion [5] suggesting independent networks. Therefore, this may not be an accurate measure of excitation of cerebellar regions for nociception.

### 1.3. How Might Neurostimulation Targeting Be Made More Reliable?

Modelling exact current changes induced by neurostimulation is also problematic. There is great anatomical and physiological variability between individuals, and the shape of the skull or arrangement of cortical folding can alter current distribution [12]. Modelling patient’s anatomical variances could allow for individually tailored and subject-specific neurostimulation. Specificity could increase beneficial outcomes by increasing stimulation focus and ensuring that the same regions are investigated between subjects, rather than using positional approximations. It has important ramifications for the expansion of neurostimulation to non-typical populations, such as those with skull deformities or stroke-related damage, or even those diagnosed with schizophrenia [13]. Current modelling around the cerebellum can be particularly problematic due to the proximity of the foramen magnum [14].

Our aim in this paper is to address this challenge by using a systematic approach with commercially available current modelling simulation software to try to discern the optimum placement for TDCS sponges and HD-TDCS electrodes when aiming to target the cerebellum. We will be focusing modelling on one lobe of the cerebellum, to align better with the literature. By rating multiple models at different locations and different current strengths, we will be able to comparatively analyse the targeting and specificity of multiple modalities. By methodically comparing and eliminating different models and options, our goal is to hone in on a modality with the most promise for focused targeting of a single cerebellar lobe.

We aim to address the following questions:

1. How much of an effect does the placement of a saline-soaked sponge cathode have on the current changes caused by ctDCS, as predicted by modelling software?
2. How focal is ctDCS predicted to be using modern current modelling software?
3. Is ctDCS affecting multiple brain regions, as predicted by modelling software?
4. How much of an effect does the placement of the central anode have on the current changes caused by HD-ctDCS, as predicted by modelling software?
5. Does amplitude change affect the current changes in ctDCS and HD-ctDCS in a visible way? How much of an effect does the placement of a saline-soaked sponge cathode have on the current changes caused by ctDCS, as predicted by modelling software?
6. Are more focal effects predicted for ctDCS or HD-ctDCS by modelling software?
7. Do current modelling predictions support the findings of research literature?
8. Is the application ctDCS and HD-ctDCS supported as having potential for influencing the experience of pain according to current modelling?

Our hypothesis is that as we systematically examine different Montages, the current models generated will highlight that certain positioning of the electrodes will activate undesirable regions of the brain that could confound the findings of cerebellar tDCS, and that there will be a Montage that is favourable for use to target specific cerebellar regions, potentially for use in analgesia studies. Additionally, we hypothesised that HD-tDCS Montages will be more focal and less likely to produce an electrical field in extracerebellar areas than a conventional tDCS Montage.

## 2. Materials and Methods

**Figure 2:**
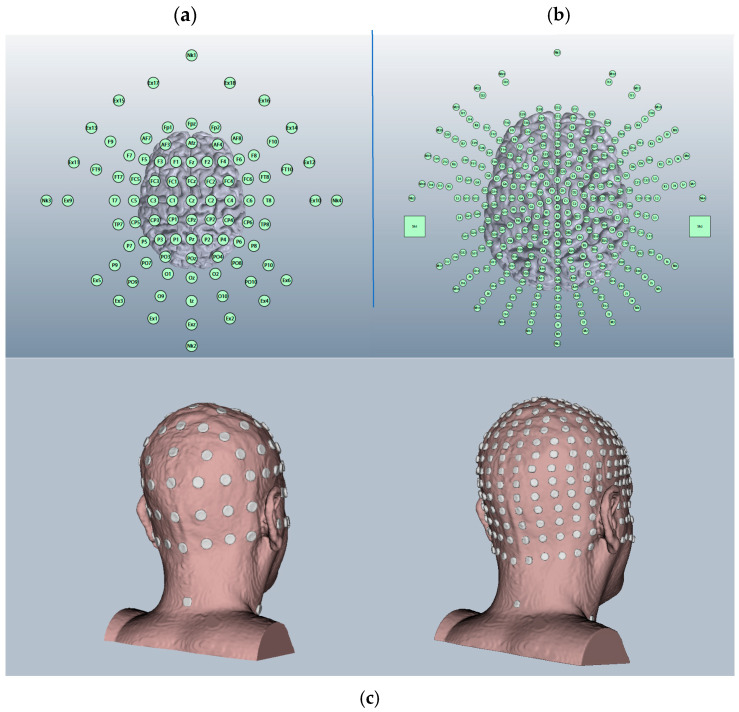
Two-dimensional and three-dimensional representations of the simulated electrode maps for Adult Male Head 1 available in Soterix software HD-Explore (2018). (**a**) The 93-electrode map used to simulate HD-tDCS; (**b**) the 338-electrode maps used to simulate tDCS; and (**c**) both maps applied to a 3D model of Adult Male Head 1.

We used Soterix HD-Explore^TM^ (Ver. 2019) brain current modelling software (2018) to systematically generate 3D models and planar cross sections (coronal, sagittal, and axial) of the right hemisphere of the cerebellum when exposed to ctDCS and HD-ctDCS at amplitudes of 1.0 mA, 1.5 mA, and 2.0 mA. We systematically evaluated current spread and affected regions based on the software’s included field intensity spectrum and visual image assessments to ascertain what appears to be the most accurate location, current strength, and tDCS/HD-tDCS modality to ensure accurate targeting of a single cerebellar hemisphere and a reduction in risk of activation of neighbouring or adjacent brain regions. Images were accessed at multiple sensitivities, from 0.1 to 0.2 V/m. We used the Adult Male Head 1 model for all generated modalities and currents to ensure comparison was possible (Figure 2).

### 2.1. Conventional ctDCS

For best comparison with previous research findings the anodal location for the conventional tDCS Montages (using a 5 cm × 5 cm electrode in a 5 cm × 7 cm saline soaked sponge) was set as over the right cerebellar hemisphere, with the centre point placed 3 cm laterally to the inion. Cathodal location outcomes were compared using the deltoid muscle, buccinator muscle, and supraorbital area as potential sites. The 338-electrode map was used for these simulations as they offered the most accurate reflection of the measurements on the saline soaked sponge.

### 2.2. High-Definition (HD) ctDCS

For the HD-tDCS modelling, four Montages were generated based on a 93 electrode point map. The 93-point map was chosen over the 338-point map that is also available as it was better reflective of the HD-tDCS equipment available in our lab. The base width of the Ag/AgCl electrode holders available with the NeuroConn HD-tDCS generator we use is 2.5 cm. Therefore, in a 5 electrode, 4 × 1 array there is not enough space available to use a 338-electrode map placement without risking bridging of current between electrodes. The locations for the four Montages are shown in Table 1 below.

## 3. Results

### 3.1. Anode and Cathode Positioning

**Figure 3:**
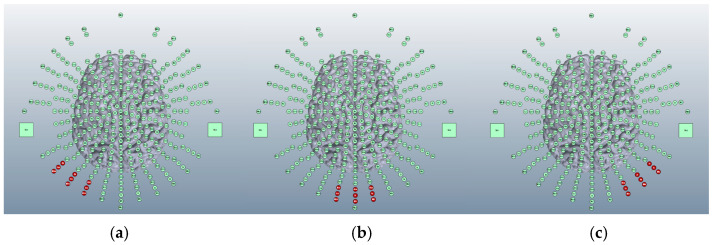
Image shows potential anodal locations generated on the 338-electrode map for Adult Male Head 1 on Soterix HD-Explore software. From left to right, (**a**) anode placed over the left cerebellar lobe, (**b**) over the cerebellar vertex (for a bilateral effect), and (**c**) over the right cerebellar lobe. Electrode positions highlighted in red represent anodal positioning.

Using a 5 × 5 cm electrode inside a 5 × 7 cm saline-soaked sponge, there are three locations in research literature for an anode aiming to target the cerebellum: over the left cerebellar lobe [15], over the right cerebellar lobe [16], or centrally to elicit a bilateral effect [17] (Figure 3).

**Figure 4:**
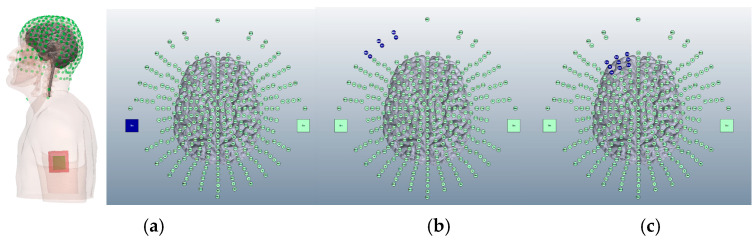
Potential cathodal sponge locations generated on the 338-electrode map for Adult Male Head 1 on Soterix HD-Explore software (2018). Shown from left to right, (**a**) the left shoulder deltoid muscle (shown both on a full upper body model and a 338-electrode map), (**b**) the left buccinator muscle, and (**c**) the left supraorbital region. Electrode positions highlighted in blue represent cathodal positioning.

Equally, there are three locations commonly used in the research literature for the cathodal electrode: the deltoid muscle [17], the buccinator muscle [18], and supraorbital area [19] (Figure 4).

For the purposes of this exploration and to keep in step with the research literature, we chose to simulate anodal stimulation of the right cerebellar lobe [16,18,19].

#### 3.1.1. ctDCS Cathode Location

Images were generated sequentially using the three available tDCS cathodal locations provided within Soterix (2018) modelling software, on the buccinator muscle, on the supraorbital area, or on the deltoid muscle. Images were generated for outcomes placing the cathode on both the left and right of each location. All initial images were set at a current of 2 mA to show the strong current effect outcomes.

##### Buccinator Cathode

**Figure 5:**
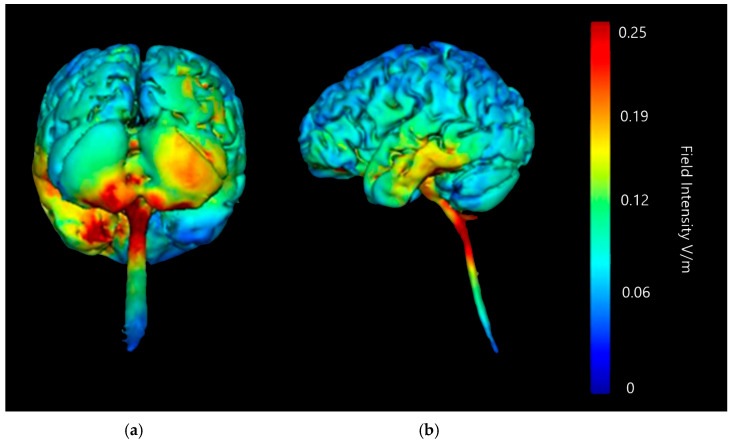
Current flow model generated simulating a 5 × 7 cm saline-soaked sponge electrode over the right cerebellar lobe (over electrode positions B12, J1, J2I3, I4, I5, M3, M4, and M5 on a 338-electrode map) and a left buccinator muscle cathode (over electrode positions M21, M22, M23, I21, I22, and I23). From left to right, (**a**) dorsal view (showing cerebellum, occipital lobe, and brainstem) and (**b**) left lateral view (showing whole brain), Field intensity V/m included for reference; image generated at 0.25 V/m.

**Figure 6:**
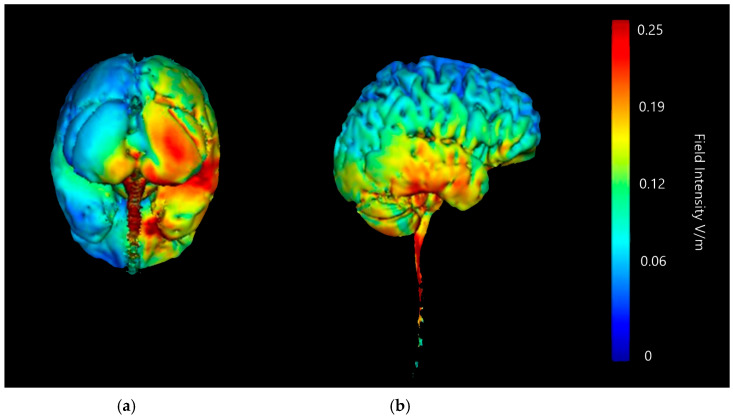
Current flow model generated simulating a 5 × 7 cm saline-soaked sponge electrode over the right cerebellar lobe (over electrodes B12, J1, J2I3, I4, I5, M3, M4, and M5 on a 338-electrode map) and a right buccinator muscle cathode (over electrodes M12, M11, M10, I12, I11, and I10). From left to right, (**a**) dorsal view (showing cerebellum, occipital lobe, and brainstem), and (**b**) right lateral view (showing whole brain). Field intensity V/m included for reference; image generated at 0.25 V/m.

Three-dimensional current flow models were generated at a strength of 2 mA with an anodal saline-soaked sponge electrode over the right cerebellar lobe and a return cathode placed over either the right or left buccinator (cheek) muscle (Figure 5 and Figure 6). Models generated showed that 2 mA of stimulation showed a high amount of current diffusion down into the upper spinal cord and the lower brainstem whether the cathode was placed on the left or the right side of the head. This would potentially cause additional activation or influence on areas such as the PAG (implicated in descended modulation of pain [20]). When examining current flow using a left buccinator placement at 2 mA, high current flow changes are highlighted in the lower part of the cerebellum bilaterally, with additional more minor current changes in the centre of the right cerebellar lobe. Examining the current flow when using the right buccinator as the cathodal site, a more consistent and strong change in the right lobe of cerebellum is present, and minimal impact is shown on current flow across the left cerebellar lobe. Both setups of the cathode on the buccinator muscles show wide diffusion of current in the parietal lobes and even some potentially significant changes in the frontal lobe, both of which are extremely undesirable and could interfere with findings of a targeted neurostimulation paradigm.

##### Supraorbital Cathode

**Figure 7:**
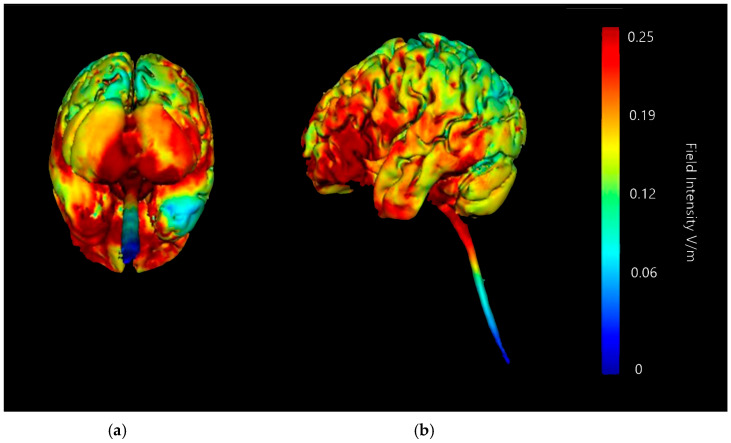
Current flow model generated simulating a 5 × 7 cm saline-soaked sponge electrode over the right cerebellar lobe (over electrode positions B12, J1, J2I3, I4, I5, M3, M4, and M5 on a 338-electrode map) and a left supraorbital region cathode (over electrode positions F10, E29, E28, F9, E30, E27, F8, E31, and E26). From left to right, (**a**) dorsal view (showing cerebellum, occipital lobe, and brainstem), and (**b**) left lateral view (showing whole brain). Field intensity V/m included for reference; image gen-erated at 0.25 V/m.

**Figure 8:**
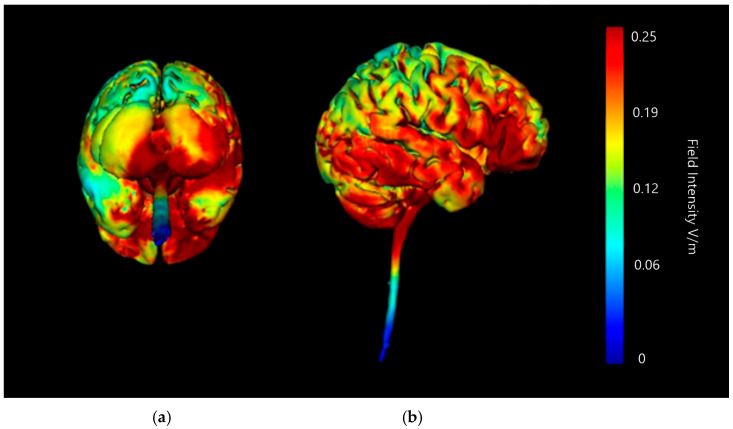
Current flow model generated simulating a 5 × 7 cm saline-soaked sponge electrode over the right cerebellar lobe (over electrode positions B12, J1, J2I3, I4, I5, M3, M4, and M5 on a 338-electrode map) and a right supraorbital region cathode (over electrode positions E11, D32, D24, and E10. D31, D25, E9, D30, and D26). From left to right, (**a**) dorsal view (showing cerebellum, occipital lobe, and brainstem), and (**b**) right lateral view (showing whole brain). Field intensity V/m included for ref-erence; image generated at 0.25 V/m.

Three-dimensional current flow models were generated at a strength of 2 mA with an anodal saline-soaked sponge electrode over the right cerebellar lobe and a return cathode placed over either the right or left supraorbital region (Figure 7 and Figure 8). Models generated showed that 2 mA of stimulation with both the left and right supraorbital region return cathode caused a high amount of current diffusion across the brain, with particularly strong changes in current intensity indicated across the parietal and frontal lobes. There are clear hotspots in parts of the frontal lobe on the left set up, and frontal and large portions of the parietal in the right set up. Both set ups also show intense current changes to the brainstem and areas such as the PAG, a concern due to its involvement in the down regulation of pain. While this may not necessarily be a negative in terms of producing analgesic outcomes, the aim of this modelling approach is to isolate effects to purely the right cerebellar lobe, so this is counterproductive. Strong changes are shown with both left and right cathode placements to the lower portions of the cerebellum; in both left and right cathode placements the current changes are bilateral. There are some lower intensity changes highlighted on the upper portion of the right cerebellar lobe, but generalised changes are shown bilaterally and are, therefore, to be considered poorly focused.

##### Deltoid Cathode

**Figure 9:**
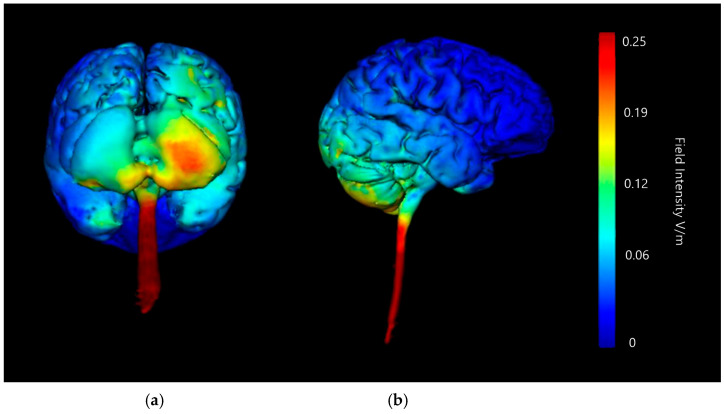
Current flow model generated simulating a 5 × 7 cm saline-soaked sponge electrode over the right cerebellar lobe (over electrode positions B12, J1, J2I3, I4, I5, M3, M4, and M5 on a 338-electrode map) and a right shoulder/deltoid muscle cathode (over electrode position Sh2). From left to right, (**a**) dorsal view (showing cerebellum, occipital lobe, and brainstem), and (**b**) right lateral view (showing whole brain from the right-hand side). Field intensity V/m included for reference; image generated at 0.25 V/m.

**Figure 10:**
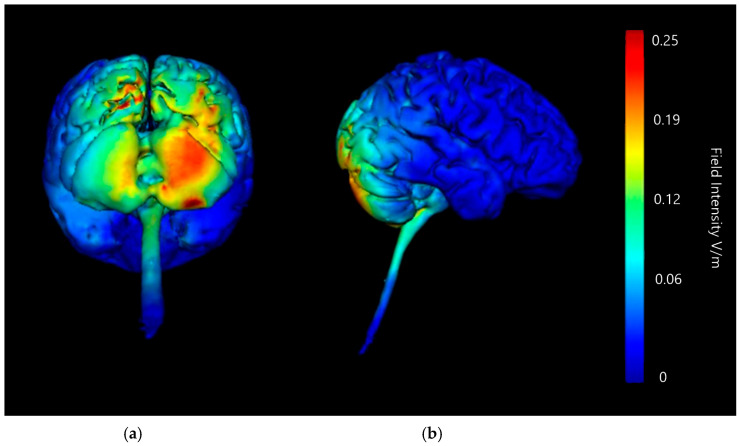
Current flow model generated simulating a 5 × 7 cm saline-soaked sponge electrode over the right cerebellar lobe (over electrode positions B12, J1, J2I3, I4, I5, M3, M4, and M5 on a 338-electrode map) and a left shoulder/deltoid muscle cathode (over electrode position Sh1). From left to right, (**a**) dorsal view (showing cerebellum, occipital lobe, and brainstem), and (**b**) right lateral view (showing whole brain from the right-hand side). Field intensity V/m included for reference; image generated at 0.25 V/m.

Three-dimensional current flow models were generated at a strength of 2 mA with an anodal saline-soaked sponge electrode over the right cerebellar lobe and a return cathode placed over either the right or left deltoid (shoulder) muscle (Figure 9 and Figure 10). The models generated showed that 2 mA of stimulation using the right deltoid muscle as a return cathode led to intense current changes down the spinal cord canal and little to no effect on the major lobes of the brain. There is a risk this movement of current could be influencing the lower portions of the brainstem, which could produce confounding effects and results outside of the targeted region. Alternatively, models generated showed that 2 mA of stimulation using the left deltoid muscle as a return cathode appears to reduce this diffusion into the spinal cord or near the brainstem noticeably. Both the left and right deltoid cathode placements indicate the probability of minor current changes in the occipital lobe, but no further diffusion into the brain, which limits the chance of influencing regions not targeted. Both left and right deltoid cathode placements show focused changes to current flow in the right cerebellar lobe, though more intense changes are shown in the left deltoid cathode set up. Conversely, the right deltoid cathode placement shows less diffusion into the left cerebellar lobe, but diffusion in both setups is shown to be minimal compared to other potential current changes.

#### 3.1.2. Image Sensitivity of Cathode Placement Simulations

**Figure 11:**
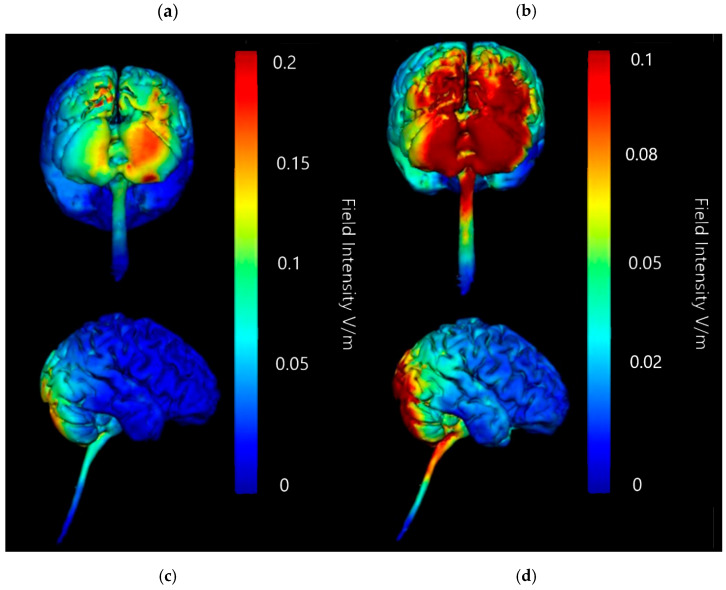
Current flow model generated simulating a 5 × 7 cm saline-soaked sponge electrode over the right cerebellar lobe (over electrode positions B12, J1, J2I3, I4, I5, M3, M4, and M5 on a 338-electrode map) and a left shoulder/deltoid muscle cathode at 2.0 mA, with adjusted image sensitivities. From left to right, (**a**) dorsal view (showing cerebellum, occipital lobe, and brainstem) at 0.2 V/m, (**b**) right lateral view (showing whole brain from the right-hand side) at 0.2 V/m, (**c**) dorsal view (showing cerebellum, occipital lobe, and brainstem) at 0.1 V/m, and (**d**) right lateral view (showing whole brain from the right-hand side) at 0.1 V/m.

**Figure 12:**
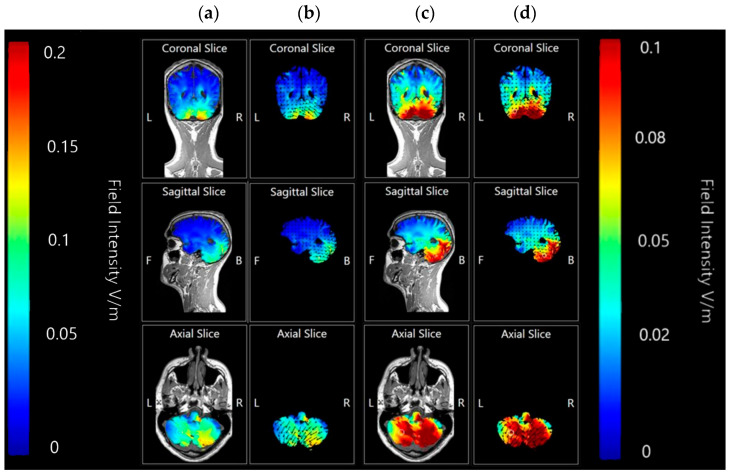
Coronal, axial, and sagittal slices of Adult Male Head 1 with simulated 2 mA applied via 5 × 7 com saline-soaked sponge anode over the right cerebellar lobe (electrode positions B12, J1, J2I3, I4, I5, M3, M4, and M5 on a 338-electrode map), and a cathode over electrode position Sh1 (left deltoid muscle). Shown in columns from left to right, (**a**) coronal, sagittal and axial slices shown as MRI style fields at 0.2 V/m sensitivity and (**b**) same slices shown as vector diagrams overlaid on the MRI style fields at 0.2 V/m, (**c**) coronal, sagittal and axial slices shown as MRI style fields at 0.1 V/m sensitivity and (**d**) same slices shown as vector diagrams overlaid on the MRI style fields at 0.1 V/m. Field intensity spectrums for each set of images have been included for reference. All slices labelled with the following: R—Right side of brain model, L—Left side of brain model, F—Front of brain model, B—back of brain model.

Simulating a 5 × 7 cm saline-soaked sponge electrode over the right cerebellar lobe (B12, J1, J2I3, I4, I5, M3, M4, and M5) and the return cathode onto the left deltoid showed localised current changes across the cerebellum without strong current changes to the upper spinal cord and brainstem (please see Table 2). To confirm simulations were in fact positing the best locations for minimal current diffusion outside the targeted area, the models for this combination (right cerebellar anode and left deltoid cathode) were regenerated with comparison images at different image sensitivities. Original images were all generated at 0.25 V/m so comparison images at 0.2 V/m and 0.1 V/m using a right cerebellar anode (B12, J1, J2I3, I4, I5, M3, M4, and M5) and a left deltoid cathode at 2 mV were generated (Figure 11 and Figure 12).

**Table 2:** Summary table of comparison of images shown in Figure 5, Figure 6, Figure 7, Figure 8, Figure 9, Figure 10, Figure 11 and Figure 12. Classification boundaries of effect using the Soterix Intensity scale present alongside Figure 5, Figure 6, Figure 7, Figure 8, Figure 9, Figure 10, Figure 11 and Figure 12 are shown in Figure 13.

Brain Areas	Cerebellar Lobe Diffusion (Left)	Cerebellar Lobe Diffusion (Right)	Spinal Cord Diffusion	Pons/ Medulla Diffusion	Occipital Lobe Diffusion (Left)	Occipital Lobe Diffusion (Right)	Parietal Lobe Diffusion (Left)	Parietal Lobe Diffusion (Right)	Temporal Lobe Diffusion	Frontal Lobe Diffusion (Left)	Frontal Lobe Diffusion (Right)	Conclusion
Deltoid anode (left)	Low	High	Low	Low	Medium	Medium	None of note	None of note	None of note	None of note	None of note	Desirable
Deltoid anode (right)	Low	High	Very High	Low	None of note	Low	None of note	None of note	None of note	None of note	None of note	Undesirable
Buccinator anode (left)	Medium	Low	Very High	Medium	Low	Low	Medium	None of note	None of note	Low	None of note	Undesirable
Buccinator anode (right)	Low	High	High	Medium	None of note	Medium	None of note	High	None of note	None of note	Medium	Undesirable
Supra orbital (left)	High	Very High	Very High	Very High	Medium	Medium	High	Medium	Medium	Very High	Very High	Undesirable
Supra orbital (right)	High	Very High	Very High	Very High	Medium	Medium	Low	Very High	Medium	Very High	Very High	Undesirable

When regenerated at higher sensitivities, simulations of current change continue to support the combination of right cerebellar lobe anode and left deltoid cathode as well targeted. Both 0.2 V/m and 0.1 V/m sensitivities show that current change remains focused highly on the right cerebellar lobe. There is little to no diffusion of current into any lobe other than occipital, and only at the highest sensitivity (0.1 V/m) does notable diffusion along the dorsal surface of the brainstem begin to happen, but the current changes do not appear to be widespread or penetrate deeply.

#### 3.1.3. Internal Views—Coronal, Sagittal and Axial

Coronal, sagittal, and axial plane slice models were also generated at 0.2 V/m and 0.1 V/m to investigate the internal effects movements of current flow through the simulated model and whether it would mirror the appearance of direction and intensity as seen on the surface of the brain model, or whether current changes might penetrate further than previously considered (Figure 12).

Slice images generated at both 0.2 V/m and 0.1 V/m sensitivity show most current flow and current intensity changes to be focused in the tissue of the cerebellum, particularly in the right lobe. The 0.1 V/m intensity highlights some minor changes current changes appearing on the coronal axis towards the first and second ventricles, although these changes may be accounted for by the high conductivity of CSF. The sagittal axis slice shows that the internal current changes have not penetrated past the cerebellar tissue and outer occipital lobe, leaving most of the brain unaffected as would be hoped for in a targeted approach. Finally, the axial slice shows that penetration into the cerebellum is thorough and mostly focused on the right side, again as targeted.

#### 3.1.4. HD-ctDCS Anode and Cathode Montages

**Figure 14:**
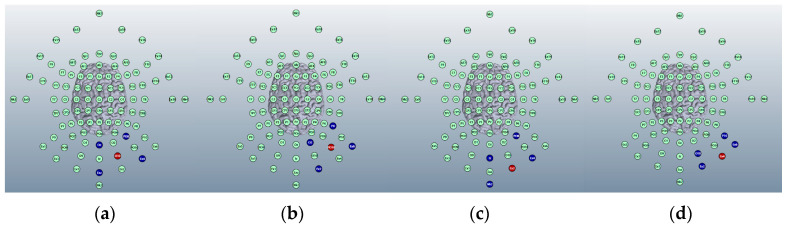
The image shows proposed potential montage locations generated on the 93-electrode map for Adult Head 1 on Soterix HD-Explore (2018) software. From left to right, montages 1–4. (**a**) Montage 1: anode placed over position O10, cathodes over positions Oz, PO8, Exz and Ex4. (**b**) Montage 2: anode placed over position PO10, cathodes over positions O2, P8, Ex2 and Ex6. (**c**) Montage 3: anode placed over position Ex2, cathodes over positions Iz, Nk2, PO8 and Ex4. (**d**) Montage 4: anode placed over position Ex4, cathodes over positions O10, P10, Ex2 and Ex6.

For HD-ctDCS five Ag/AgCl electrodes are used in a standard 4 × 1 pattern, with the central electrode polarity determining the type of treatment administered (anodal or cathodal). As the right cerebellar lobe has already been selected for the anode in our ctDCS Montage, it follows that for comparison of targeting outcomes the central electrode for HD-ctDCS was also be simulated here. The central electrode for HD-ctDCS is smaller (12 mm) than the 5 × 7 cm sponge anode used in ctDCS so finer targeting may be possible. We identified four positions on the 93-electrode map over the right cerebellar lobe that could operate as the centre of a 4 × 1 electrode Montage: O10, PO10, Ex2 and Ex4. These are shown in Figure 14.

For initial comparison, images were generated using our four Montages at a current of 2 mA and field intensity threshold of 0.2 V/m. For side-by-side comparisons of all four Montages please see Table 3.

##### Montage 1: Anode O10, Cathodes Oz, PO8, Exz, and Ex4

**Figure 15:**
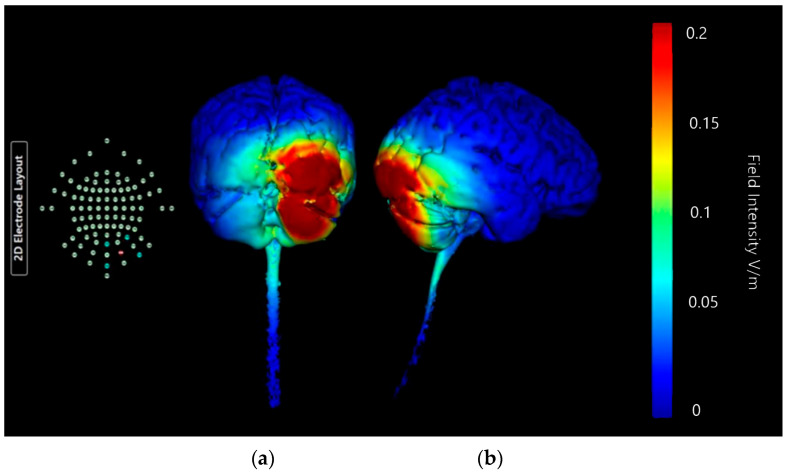
Three-dimensional current flow model generated simulating an anodal electrode at location O10 and cathodal electrodes at locations Oz, Exz, PO8, and Ex4 using 2.0 mA of current. From left to right: (**a**) dorsal view (showing cerebellum, occipital lobe, and brainstem), and (**b**) right lateral view (showing whole brain from the right-hand side). Field intensity V/m included for reference; image generated at 0.2 V/m.

Montage 1 shows a strong change in current specifically on the right lobe of cerebellum and the right occipital lobe (Figure 15). There is no notable current diffusion to other lobes or the brainstem. The central focus of the most intense current changes in this Montage appears to be in the junction between cerebellum and occipital lobe, which is not the desired target. However, the right cerebellar lobe does show intense current changes across its surface, with the influence limited to the right-hand side, suggesting potential for this Montage.

##### Montage 2: Anode PO10, Cathodes O2, P8, Ex2, and Ex6

**Figure 16:**
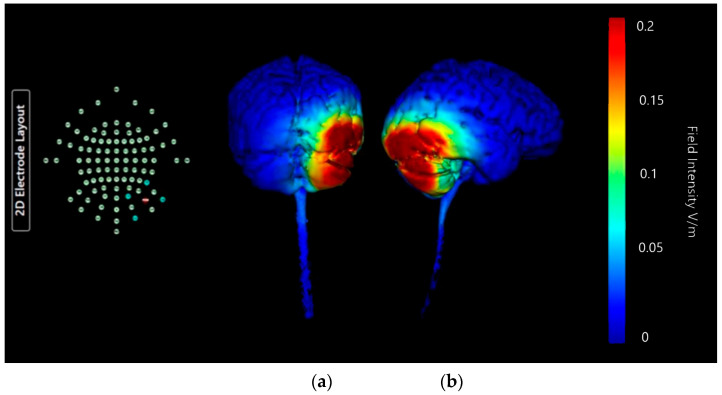
Three-dimensional current flow model generated simulating an anodal electrode at location PO10 and cathodal electrodes at locations O2, Ex2, P8, and Ex6, using 2.0 mA of current. From left to right, (**a**) dorsal view (showing cerebellum, occipital lobe, and brainstem), and (**b**) right lateral view (showing whole brain from the right-hand side). Field intensity V/m included for reference; image generated at 0.2 V/m.

Montage 2 shows a similar strong and specific effect on right lobe of cerebellum and right occipital lobe, though the focal point of the effect is more lateral and appears to cross into the parietal lobe as well (Figure 16). There is no notable current diffusion to the temporal or frontal lobe, and no diffusion into the brainstem. All induced current changes remain limited to the right-hand side. When compared to Montage 1 the central focus of the current changes is lower on the coronal plane and a higher share of overall current changes is contained in cerebellar tissue. The possible diffusion across lobes in this Montage is undesirable, but it is well focused on the cerebellum so may still have potential application.

##### Montage 3: Anode Ex2, Cathodes Iz, Nk2, PO8, and Ex4

**Figure 17:**
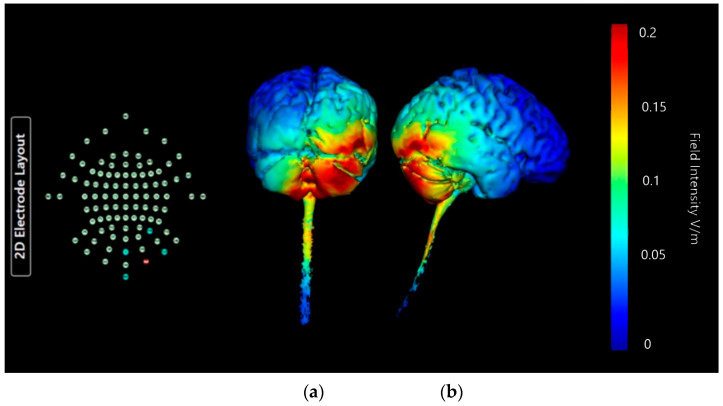
Three-dimensional current flow model generated simulating an anodal electrode at location Ex2 and cathodal electrodes at locations Iz, Nk2, PO8, and Ex4, using 2.0 mA of current. From left to right, (**a**) dorsal view (showing cerebellum, occipital lobe, and brainstem), and (**b**) right lateral view (showing whole brain from the right-hand side). Field intensity V/m included for reference; image generated at 0.2 V/m.

Montage 3 produces widespread intense current changes in the right cerebellar lobe, but they are no longer limited to the one side (Figure 17). Current diffusion and changes in the left cerebellar lobe, and changes present throughout the vermis are also shown. There are minor current changes present in the lower occipital lobe, although not as intense or as widespread as in Montages 1 or 2. However, it is important to note that current diffusion into the brainstem and spinal cord is clearly indicated, which is highly undesirable. Therefore, when considering that targeting the right cerebellar lobe is the goal, the current shunting into the left cerebellar lobe and upper brainstem do indicate that it would be hard to discern if any influence of neurostimulation were due to effects on the right cerebellar lobe, or in fact produced by current changes in adjacent regions. Due to this, although this Montage shows a significant effect on the right cerebellar lobe and limited occipital lobe changes, it is unusable.

##### Montage 4: Anode Ex4, Cathodes O10, P10, Ex2, and Ex6

**Figure 18:**
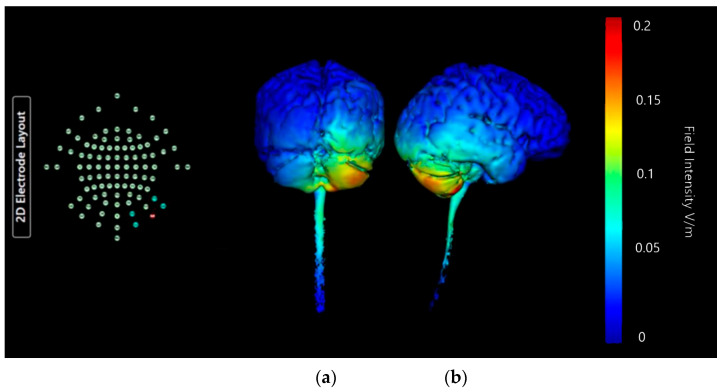
Three-dimensional current flow model generated simulating an anodal electrode at location Ex4 and cathodal electrodes at locations O10, Ex2, P10, and Ex6, using 2.0 mA of current. From left to right, (**a**) dorsal view (showing cerebellum, occipital lobe, and brainstem), and (**b**) right lateral view (showing whole brain from the right-hand side). Field intensity V/m included for reference; image generated at 0.2 V/m.

Montage 4 shows a small, strong current effect centrally in the right cerebellar lobe (Figure 18). The distribution of current intensities in this image suggests most of the effect of the current change may be internal and would require generation of axial, sagittal, and coronal slices of the above model to verify. There are some weak current changes highlighted on edges of the occipital lobe, and near the top of the spinal canal. All current changes and effects remain limited to the right-hand side of the model. Though the current changes seen in this Montage are weaker than in Montages 1, 2, or 3, they are the most focused on the single right cerebellar lobe with minimal additional influence, which is the goal.

#### 3.1.5. Choice of HD-ctDCS Montage

For a side-by-side comparison of the four proposed HD-ctDCS Montages and how they affected current changes please see Table 3. Of the four proposed set ups, we decided to progress with further investigation into Montages 2 and 4. Montage 4 was selected as it showed the most focal effects on the right lobe of the cerebellum; however, the current effects are weak and may be unlikely to produce significant results in an experimental setting, leading to the need for further checks. Montage 2 was selected as it showed strong and thorough current effects on the targeted right cerebellar lobe, but diffusion into surrounding areas like the occipital lobe which should be assessed for likelihood of additional activations. Importantly, both show no effects on the rest of the cerebellum or on the brainstem.

##### Comparing Montage 2 (Anode PO10) and Montage 4 (Anode Ex4): Increased Image Sensitivity

**Figure 19:**
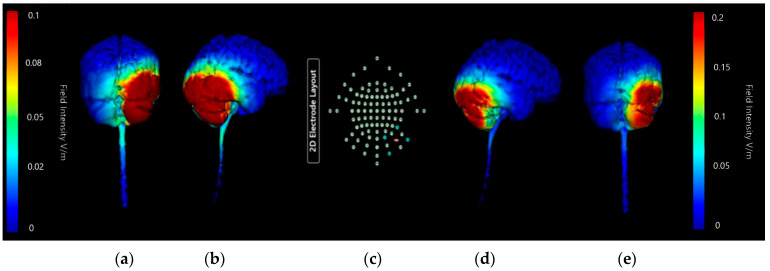
Three-dimensional current flow model generated simulating an anodal electrode at location PO10 and cathodal electrodes at locations O2, Ex2, P8, and Ex6, using 2.0 mA of current, with increased image sensitivity. From left to right, (**a**) dorsal view (showing cerebellum, occipital lobe, and brainstem), at 1.0 Vm and (**b**) right lateral view (showing whole brain from the right-hand side) at 1.0 V/m, (**c**) 93-electrode map with anodal position highlighted in red and cathodal position highlighted in blue, (**d**) dorsal view (showing cerebellum, occipital lobe and brainstem), at 2.0 Vm and (**e**) right lateral view (showing whole brain from the right- hand side) at 2.0 V/m. Field intensity V/m included for reference.

**Figure 20:**
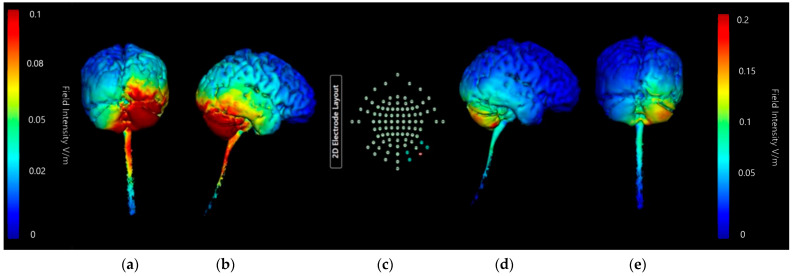
Three-dimensional current flow model generated simulating an anodal electrode at location Ex4 and cathodal electrodes at locations O10, Ex2, P10, and Ex6, using 2.0 mA of current, with increased image sensitivity. From left to right: (**a**) dorsal view (showing cerebellum, occipital lobe, and brainstem), at 1.0 Vm and (**b**) right lateral view (showing whole brain from the right-hand side) at 1.0 V/m, (**c**) 93-electrode map with anodal position highlighted in red and cathodal position highlighted in blue, (**d**) dorsal view (showing cerebellum, occipital lobe and brainstem), at 2.0 Vm and (**e**) right lateral view (showing whole brain from the right-hand side) at 2.0 V/m. Field intensity V/m included for reference.

Montage 2 (Figure 19) and Montage 4 (Figure 20) models were regenerated at a higher image sensitivity of 0.1 V/m for further comparison.

**Figure 21:**
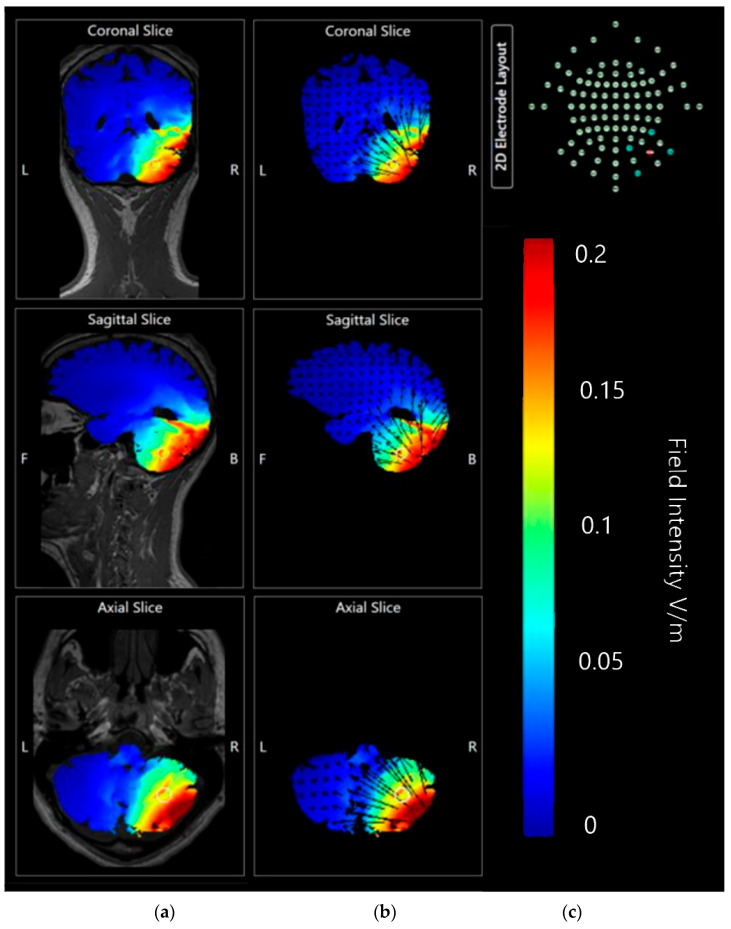
Coronal, axial, and sagittal slices of Adult Male Head 1 with simulated 2 mA applied via anodal electrode at location PO10 and cathodal electrodes at locations O2, Ex2, P8, and Ex6 on a 93- electrode map. Shown in columns from left to right, (**a**) coronal, sagittal and axial slices shown as MRI style fields at 0.2 V/m sensitivity and (**b**) same slices shown as vector diagrams overlaid on the MRI style fields at 0.2 V/m, and (**c**) 93-electrode map showing position of anode highlighted in red and cathodes in blue, with field intensity spectrums for all images for reference. All slices labelled with the following: R—Right side of brain model, L—Left side of brain model, F—Front of brain model, B—back of brain model.

Images generated at a field intensity of 0.1 V/m corroborate the models generated at a field intensity of 0.2 V/m. In Montage 2, this shows that the strength and intensity of the elicited current changes are equal across both the right cerebellar lobe and right occipital and parietal lobes, with a wider spread effect than was seen on initial projections. The higher sensitivity images for Montage 4 highlight that possible current effects on the brainstem and spinal cord could be created, but do confirm that most of the current changes are focused in the right lobe of the cerebellum unilaterally. There is also visible some diffusion into the parietal lobe that was not immediately evident at the lower sensitivity. While both show the potential for current influences on other regions that would be undesirable, Montage 4 again demonstrates a more focal effect with less likely diffusion than Montage 2. To confirm this, coronal, sagittal, and axial slice models were generated using Montage 4 at a current of 2 mA and a sensitivity of 0.2 V/m to investigate projected internal current flow (Figure 21).

Slice images show deep and directional current flow penetration into the tissue of the right cerebellar lobe, with no effect bilaterally as predicted by the 3D surface model. The effects of current on the occipital lobe are shown to be shallow and limited to the outer surface of the brain, making it unlikely they will have widespread effects. The changes seen away from the cerebellum may also be influenced by proximity to CSF, as the parietal changes show some interaction with the presence of the first and second ventricles.

#### 3.1.6. Summation

Several important considerations have been highlighted by modelling these Montages. Placing an anode too far from the central line means less likelihood of affecting the cerebellum alone and a higher likelihood that there will be diffusion into the parietal lobe. Placing the anode in a very low position on the electrode map makes it most likely to affect the cerebellum and not the occipital lobe. Placing the focal electrode two electrode positions away from the midline appears to make it less likely stimulation will have a bilateral effect and more likely to affect whole cerebellar lobe than placing it 1 or 3 positions away. Based on these considerations, a suggested ideal positioning would be a low spot not too far from the central rear point of the skull, but far enough away to have a lateral effect. There is approximately a 7 cm distance between the inion and the mastoid process and placing the anode roughly 3 cm laterally from the inion, as is often the case in research literature, is supported by these models [15,16,17,18].

### 3.2. Current Strength

With different types of electrode Montages for both modalities considered, the question had turned to whether different levels of current would affect targeted neurostimulation in terms of focality to its detriment. Simulations of 3D models affected by three different currents (1.0 mA, 1.5 mA, and 2.0 mA) were sequentially generated using the Montages identified as most desirable for ctDCS, the right cerebellar anode and left deltoid cathode.

#### 3.2.1. ctDCS Montage: Right Cerebellar Lobe and Left Deltoid Muscle

**Figure 22:**
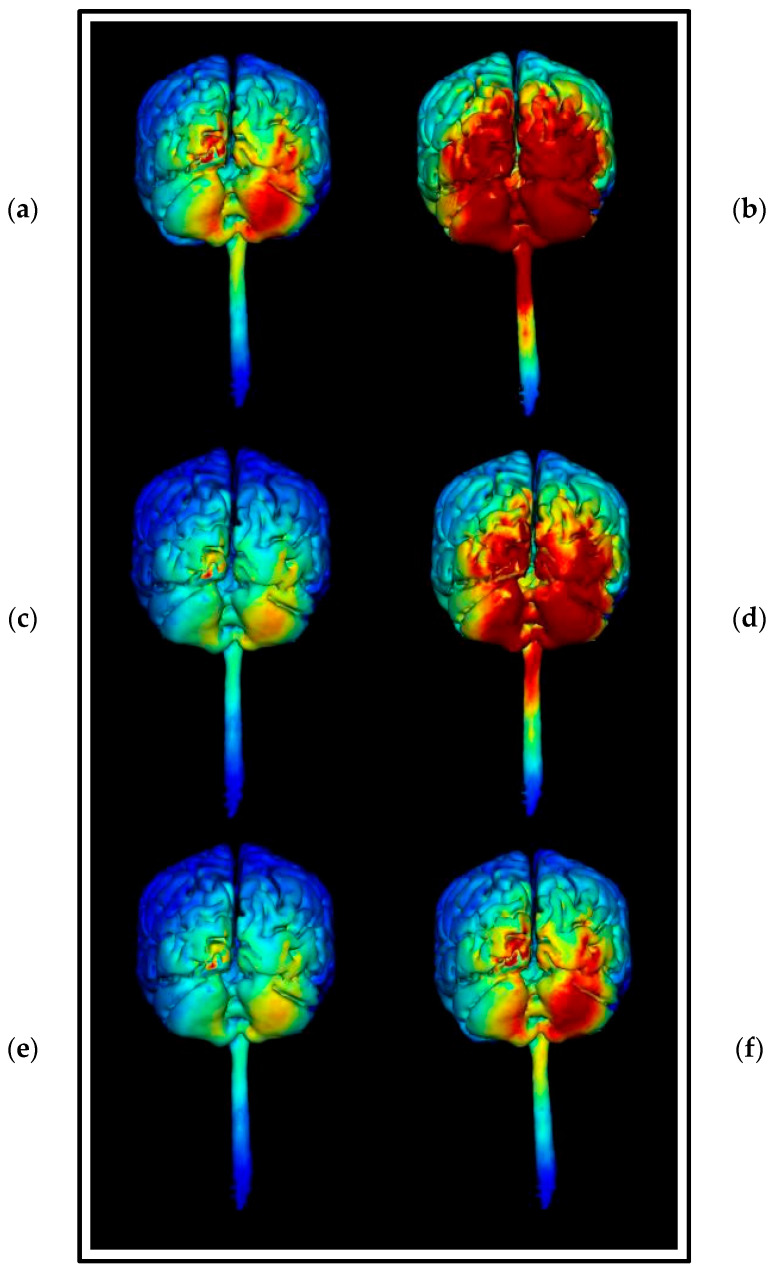
Comparison of dorsal views of 3D models generated using a 5 × 7 cm saline-soaked sponge over electrode positions B12, J1, J2I3, I4, I5, M3, M4, and M5 (on a 338-electrode map) and a return cathode over the left deltoid muscle at 2.0 milliamps, 1.5 mA, and 1.0 mA with image sensitivity adjusted. Shown from left to right, (**a**) application of 2.0 mA at 0.17 V/m sensitivity, (**b**) application of 2.0 mA at 0.08 V/m sensitivity, (**c**) application of 1.5 mA at 0.17 V/m sensitivity, (**d**) application of 1.5 mA at 0.08 V/m sensitivity, (**e**) application of 1.0 mA at 0.119 V/m sensitivity (the lowest sensitivity threshold the software would generate at, and (**f**) application of 1.0 mA at 0.08 V/m Field intensity scale not included).

An attempt was made to generate images for 1.0 mA, 1.5 mA, and 2.0 mA using the final selected ctDCS Montage (anode sponge over positions B12, J1, J2I3, I4, I5, M3, M4, and M5 on a 338-electrode map, cathode position over left deltoid muscle), all initially at a sensitivity of 0.17 V/m (software default). Changing the current to 1.0 mA made the changes across the brain too small to register with the modelling software with anything less sensitive than 0.08 V/m. Images for 1.5 mA and 2.0 mA were regenerated at 0.08 V/m sensitivity but the low threshold for registering current change made it difficult to discern differences in diffusion or current change strengths at this sensitivity with the higher currents. An example below (Figure 22) shows the 0.08 sensitivity images for 1.0 mA, 1.5 mA, and 2.0 mA, but ultimately it was decided to continue with the comparison of only 1.5 mA and 2.0 mA, as if the changes simulated at 1.0 mA were so small as to need a sensitivity of imaging changes by the software to be able to register them, it is unlikely that the effects of stimulation at this strength are going to be intense and therefore affected strongly by anodal or cathodal position.

**Figure 23:**
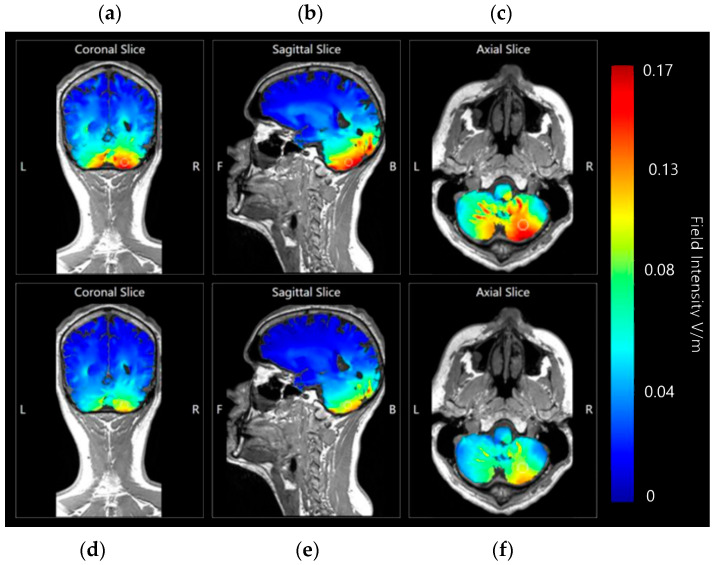
Coronal, axial, and sagittal slices of Adult Male Head 1 with simulated 1.5 mA and 2.0 mA applied over electrode positions B12, J1, J2I3, I4, I5, M3, M4, and M5 on a 338-electrode map, and with a cathode over the left shoulder muscle. Shown from left to right, (**a**) coronal slice shown as MRI style field at 0.17 V/m sensitivity and 2.0 mA of current, (**b**) sagittal slice shown as MRI style field at 0.17 V/m sensitivity and 2.0 mA of current, (**c**) axial slice shown as MRI style field at 0.17 V/m sensitivity and 2.0 mA of current, (**d**) coronal slice shown as MRI style field at 0.17 V/m sensitivity and 1.5 mA of current, (**e**) sagittal slice shown as MRI style field at 0.17 V/m sensitivity and 1.5 mA of current, (**f**) axial slice shown as MRI style field at 0.17 V/m sensitivity and 1.5 mA of current. Field intensity spectrum has been included for reference. All slices labelled with the following: R—Right side of brain model, L—Left side of brain model, F—Front of brain model, B—back of brain model.

Coronal, sagittal, and axial slices were generated and compared for effects produced by a current of 1.5 mA and 2.0 mA at 0.17 V/m image sensitivity (Figure 23).

Application at both 1.5 mA and 2.0 mA current show localised effects centred on the right cerebellar lobe, and minimal diffusion into the rest of the brain. At an image sensitivity of 0.17 V/m, the effects of 1.5 mA appear to be less intense and have only moderately affected current flow or influenced brain tissue. The 2.0 mA images, however, show a much stronger result focused in the same area.

#### 3.2.2. Summary of Findings

Previous studies that have shown significant results following tDCS or HD-tDCS have been operating at a current level of 2.0 mA [21,22,23]. Participants in these studies have reported few adverse effects following stimulation, suggesting it is a tolerable current level for most [24]. Studies at a lower 1.0 mA/1.5 mA have also produced significant results [24]; however, a higher voltage is likely to display more significant changes and outcomes following stimulation protocol.

Based on the models we simulated, and information from the research literature, we have found that to achieve a highly targeted neurostimulation protocol of the cerebellum, with minimal diffusion into surrounding areas and targeted current changes is theoretically possible. We have identified that for a ctDCS Montage, the opposite deltoid muscle to the targeted lobe and a voltage of 2.0 mA is the most focused and effective. For a targeted HD-ctDCS protocol, we have demonstrated that specific placement of the central anode can have major effects on the location stimulation is delivered to, how many surrounding regions it affects, and how targeted the penetration of current is.

## 4. Discussion

We used commercially available Soterix^®^ current modelling software to sequentially and systematically explore current diffusion simulating Montages used in cerebellar NIBS research protocols. Several important considerations were highlighted with these models. In a standard ctDCS anodal saline soaked sponge protocol, if the aim is to increase focality and targeting of specific areas of the cerebellum (as was our goal here), our models showed that anodes should not be placed too far from the central line, as it increases the likelihood of current diffusion activations in the parietal lobe. Additionally, it appears to increase focality of the cerebellum and decrease occipital lobe diffusion when the anode is placed on the lowest point of our electrode map, nearest the base of the skull. These models suggest that the most focal location is placing the anode roughly 3 cm laterally from the inion, a position used in research studies already [15,16,17,18]. The optimal placement of the cathode was on the contralateral deltoid muscle, supporting previous modelling studies that discounted the buccinator and supraorbital bone as cathode locations [13].

A HD-ctDCS simulation showed that highly focal stimulation is theoretically possible, but that even small changes in location on the central anode can potentially change the brain regions activated and cause large changes in the area affected by current diffusion. This may suggest that clear anatomical landmarks are critical for a good Neurostimulation protocol design, and future work with these protocols need to consider the inclusion of measuring cerebral landmarks carefully or using MRI for focalization. It is important that research designs consider carefully how activation of surrounding regions could affect the variables they are studying and plan the location of their electrodes carefully before starting stimulation.

### 4.1. How Is This Applicable to Pain?

There is increasing evidence of the role of the cerebellum in pain processing [5,6,7,8,25,26]. The cerebellum is thought to have a role in multiple different pathways that are activated during pain and the modulation of pain [27]. The cerebellum is likely to be involved in purely sensory, nociceptive aspects of pain, but also pain-avoidance during movement, and the coordination with autonomic areas that are associated with pain avoidance. Additionally, the cerebellum is thought to have a role in pain expectation, endogenous pain modulation pathways, placebo analgesia and networks associated with reward after removal of pain and pain aversion.

Activation of the cerebellum occurs during acute pain, and alterations in cerebellar activation is thought to occur during chronic pain in patient groups. The activation of the cerebellum in acute pain can be in both lobes or unilateral, and this is thought to be dependent on modality of stimulation (thermal vs. pressure).

Acute pain is associated with an increase in cerebellar activity, whereas chronic pain has mixed effects [28,29]. Non-invasive brain stimulation (NIBS) for pain relief is emerging as a therapeutic option and the development of novel NIBS interventions for pain modulation is an important aim given the problems associated with pharmacological pain relief. So far, the research has focused on stimulation of motor cortex, an area that was previously established to reduce pain with implanted electrodes. The pre-frontal cortex is also an area that has been considered [30,31]. Given the possible pain modulatory networks that the cerebellum is likely to contribute to, it is an obvious target for investigation for NIBS. However, there are several uncertainties in the literature regarding cerebellar stimulation. Animal research indicates that stimulation of the cerebellum can have pro and anti-nociceptive effects [32,33,34]. Additionally, cerebellar tDCS is relatively underexplored and there is less clarity regarding the appropriate Montages and stimulation protocols. Furthermore, investigations on the cellular consequences of cerebellar stimulation are fewer. The cerebellum has a much higher density of neurons as well as a higher diversity in cell types. So while they are currently understudied, they are in fact of critical importance if cerebellar stimulation is to provide useful interventions. In the current study, we used current modelling software to identify the most appropriate electrode grouping placement for HD-ctDCS that targeted a single lobe of the cerebellum (in this investigation, the right lobe) with the least amount of current diffusion into other surrounding tissues and structures, and, therefore, the highest focus on our target area. Such Montages should help to delineate the specific role of the cerebellum within pain processing and avoid stimulation of surrounding regions, that may additionally contribute to pro or anti- nociceptive effects.

### 4.2. Focality of Stimulation in HD-tDCS

The development of optimal stimulation protocols is further dependent on consideration of the stimulation ‘dosage’. Typically, tDCS stimulation ranges from 1.0 to 2.0 mA. However, there has been non-linearity in the effects [35]. Using the higher amplitude of 2.0 mA had the best outcomes in terms of tissue penetration and area of effect, even when focused in on a target area such as a single cerebellar lobe. The modelling was performed under several different current strengths and compared how these affected not only the brain surface of our model, with coronal, sagittal and axial slices generated to visualise the depth of current penetration. While diffuse current flow has been shown to be advantageous in some circumstances, for cerebellar NIBS focal stimulation is likely to be most appropriate. Our goal was to produce recommendations for highly focused, minimally extracerebellar diffusing, tDCS Montages to target an individual lobe of the cerebellum. It is vital that any electrode Montage minimises surface area targeted when attempting to link stimulation of a specific target region with a behavioural effect, and as TDCS has a traditionally low spatial resolution [36] it is of clear research interest to address focality of any modality.

Through comparison and elimination, we identified using our generated models the most focal layout that exhibited the least diffusion into areas outside the cerebellum for both a ctDCS and a HD-ctDCS protocol. This approach aids the development of protocols where NIBS can be appropriately targeted and subsequently linked to behavioural effects in experimental studies.

Our Hypothesis was that a HD-TDCS Montage would be more focal and less likely to produce and electrical field in extracerebellar areas than a conventional TDCs Montage. This was supported by the models we generated and the Montage we identified (Montage 4) as the most focal. Unexpectedly, when increasing the amplitude from 0.5 mA to 1.0 mA and 2.0 mA, it was expected that the models would show further current diffusion into surrounding areas. However, when the location of the electrodes, particularly for the HD-ctDCS had been optimised, the modelling implies that a higher amplitude allowed for better tissue penetration without compromising focality of region.

One of the major focuses of transitioning from conventional to HD tDCS for any brain area has been an attempt to improve the focality of the stimulation. In this study, we used extensive current modelling and comparisons of Montages for both forms of tDCS to ensure not only that we were successfully targeting the area we want, but that we were not accidentally stimulating a secondary area as well that might influence any findings post-stimulation. One of the areas of the brain that was identified as being at risk of secondary activation during ctDCS was the PAG. The PAG is involved in descending pain modulation. Montages for stimulation of the cerebellum were selected that had the lowest chance of influencing this area, and were as isolators of the cerebellum as possible, but we may have to accept that in being this focused and excluding the interactions of other brain structures as much as possible, our Montage may reduce the likelihood of significant neurostimulation effects on nociception. If the cerebellum is indeed an integrative area, it may well need the interactions with other pain matrix structure to have analgesic effect, in symphony rather than isolation.

### 4.3. Applications to Clinical Practice

There are two key challenges facing those trying to apply ctDCS in a clinical setting: firstly, a lack of a suitable data bank of healthy individuals undergoing ctDCS for comparison to, and secondly, a lack of consensus in research as to the protocol used [37]. There is large variation across studies regarding anodal vs. cathodal, electrode placement, voltage, or length of treatment [13,38]. Until this is addressed, results cannot be directly compared, and a standard protocol will be hard to create. The information in this paper is presented to demonstrate the importance of carefully planning a study to include discrimination between research protocols based on the topic and brain region, and to highlight the theoretical possibility of an ideal cerebellar tDCS Montage.

### 4.4. Limitations

It is a limitation of our modelling that we worked with a single projected head, based upon an adult male (Model was named “Adult Male 1”). While we can clearly show the effects of changing focality and current diffusion using different strengths, our model does not account for anatomical variation in individuals, or differences in external factors such as age or sex of an individual. Some research has suggested skull thickness differences based on ethnicity and gender differences. Li et al. [39] found that the sex of an individual significantly affected cerebellar activation as shown on an fMRI following painful stimuli [3]. They also found differences in signal intensity of cerebellar activation dependent on the type of pain (muscle vs. cutaneous) used to evoke the response. This shows that even with the best modelling available, practical application will need to account for the type of cerebellar activation being targeted by stimulation, and the individual themselves being stimulated to have a chance of increasing efficacy. Unfortunately, these factors are outside the scope of this paper.

Klaus et al. [36] carried out a similar comparison of electric field distribution on 20 head models at 2 mA using the ipsilateral buccinator muscle and contralateral supraorbital area as placements for the return electrode in a conventional tDCS set up. For comparison, we found through our modelling that the contralateral deltoid muscle was the optimum placement for the anode. Klaus et al. did not use the deltoid muscle, but their findings using the buccinator and supraorbital region did support the use of smaller electrodes (such as in HD-TCDS) in close vicinity of the targeted neural area to reduce extracerebellar electrical fields. They concluded that using smaller electrodes placed closely over the target area could significantly reduce extracerebellar electrical fields.

A further systematic evaluation of the effects of ctDCS on cerebellar–brain inhibition [37] found that the position of the return electrode of ctDCS has no significant impact of post-ctDCS physiological effects. Therefore, it is possible our modelling outcomes and those of Klaus et al. [36] could be used to refine the target even further through comparison, perhaps a target for further investigation.

### 4.5. Future Directions

The findings of this investigation and the suggested HD-ctDCS Montage are all theoretical, and the next step would be to compare outcomes in inducing analgesia using ctDCS using different orientations and positions of the stimulation electrodes on both pain threshold with differing modalities, and pain modulation. Investigating whether higher focality on the cerebellum increases the likelihood of an analgesic response to stimulation, or whether the further diffusion of current is more effective, can help add to our understanding of the amount the cerebellum controls nociceptive response in the pain matrix. Additionally, comparing analgesic effects on different pain threshold tasks (such as comparing Heat–Pain threshold and Pressure–Pain threshold tasks, for example) may show whether discrimination of different pain types has an influence on the cerebellum’s role. As it is often argued that the cerebellum is responsible for aiding in motor responses to pain stimuli, it could be interesting to further investigate if the difference in stimuli produces different outcomes following neurostimulation.

Alternatively, using structural imaging via MRIs to map an individual participants anatomy could additionally help refine the use of ctDCS (indeed, TCDS in general) as a treatment modality, and inform improvements in modalities for research. However, given the costs associated with this approach it would be beneficial if modelling current flow with reference to a range of standardised scans would provide sufficiently efficacious results.

Additionally, considering the effects of polarity of the effectiveness of tDCS could be an interesting avenue of investigation. It has not been clearly demonstrated that anodal stimulation would be the optimum for a cerebellar neuro-stimulation protocol [18].

## 5. Conclusions

The cerebellum is an important novel target region for cerebellar NIBS. For cerebellar NIBS to be optimised, standardised electrode Montages need to be developed to produce optimised analgesic effects. This study provides the first step at characterising differences in Montages used in the literature, highlighting the differences in current flow that will occur in these protocols that have previously not been considered in designing ctDCS for pain. These differences will likely underlie the current variability observed in efficacy of cerebellar NIBS.

## Figures and Tables

**Figure 13:**
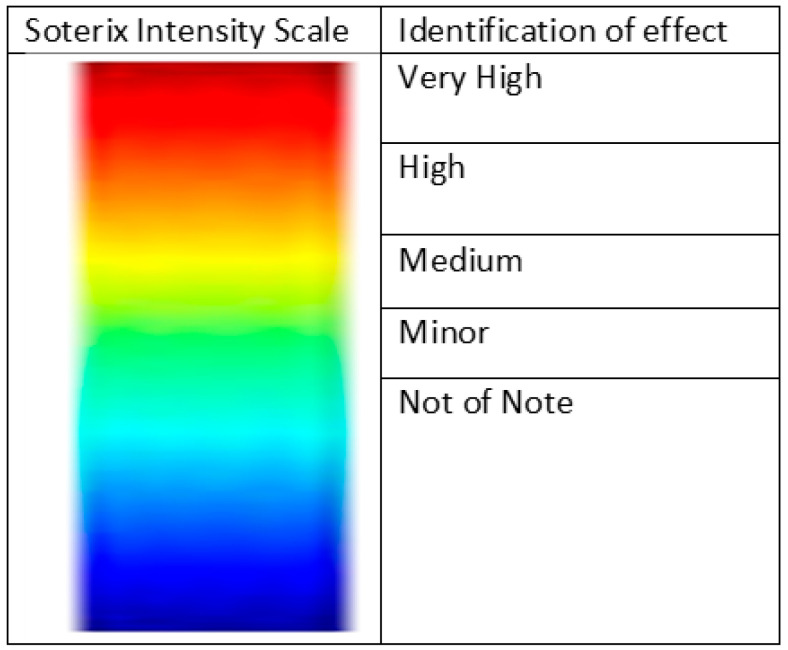
Classifications of effect strength shown on generated Soterix images to portray intensity or current changes during neurostimulation protocols.

**Table 1:** Summary of anodal and cathodal placements on a 93-electrode map 3D simulation for four HD-ctDCS montages as generated in Soterix HD-Explore (2018).

Montage	Anode Position	Cathode Positions
1	O10	Oz
		PO8
		Exz
		Ex4
2	PO10	O2
		P8
		Ex2
		Ex6
3	Ex2	Iz
		NK2
		PO8
		Ex4
4	Ex4	O10
		P10
		Ex2
		Ex6

**Table 3:** Summary table of comparison of images shown in Figure 15, Figure 16, Figure 17 and Figure 18. Classification boundaries of effect using the Soterix Intensity scale present alongside Figure 15, Figure 16, Figure 17 and Figure 18 are shown in Figure 13.

	Cerebellar Lobe Diffusion (Left)	Cerebellar Lobe Diffusion (Right)	Spinal Cord Diffusion	Pons/ Medulla Diffusion	Occipital Lobe Diffusion (Left)	Occipital Lobe Diffusion (Right)	Parietal Lobe Diffusion (Left)	Parietal Lobe Diffusion (Right)	Temporal Lobe Diffusion	Frontal Lobe Diffusion (Left)	Frontal Lobe Diffusion (Right)	Conclusion
Montage 1: Anode O10	None of note	High	None of note	None of note	None of note	Very High	None of note	None of note	None of note	None of note	None of note	Potential
Montage 2: Anode PO10	None of note	High	None of note	None of note	None of note	High	None of note	Low	None of note	None of note	None of note	Potential
Montage 3: Anode Ex2	High	High	Medium	Medium	Low	High	None of note	Low	Low	None of note	None of note	Undesirable
Montage 4: Anode Ex4	Very Low	Medium	None of note	None of note	None of note	Low	None of note	Low	None of note	None of note	None of note	High Potential

## Data Availability

Data are contained within the article.

## Abbreviations

The following abbreviations are used in this manuscript:

tDCS	Trans-cranial Direct Current Stimulation
ctDCS	Cerebellar Trans-cranial Direct Current Stimulation
HD-ctDCS	High-Definition Cerebellar Trans-cranial Direct Current Stimulation
NIBS	Non-Invasive Brain Stimulation
PAG	Peri-aqueductal Grey
EEG	Electroencephalogram
IASP	International Association for the Study of Pain
MRI	Magnetic Resonance Imaging
fMRI	Functional Magnetic Resonance Imaging
PET	Positron Emission Topography
EMG	Electromyogram
mA	Milli-Amps
V/m	Volt per Metre
cm	Centimetre
Ag	Silver
AgCl	Silver Chloride
2D	Two-Dimensional
3D	Three-Dimensional

## References

[1] Merskey H., Bogduk N. (2021). Classification of Chronic Pain.

[2] Raja S.N., Carr D.B., Cohen M., Finnerup N.B., Flor H., Gibson S., Keefe F.J., Mogil J.S., Ringkamp M., Sluka K.A. (2020). The revised International Association for the Study of Pain definition of pain: Concepts, challenges, and compromises. Pain.

[3] Moulton E.A., Schmahmann J.D., Becerra L., Borsook D. (2010). The cerebellum and pain: Passive integrator or active participator?. Brain Res. Rev..

[4] Baumann O., Borra R.J., Bower J.M., Cullen K.E., Habas C., Ivry R.B., Sokolov A.A. (2015). Consensus Paper: The Role of the Cerebellum in Perceptual Processes. Cerebellum.

[5] Borsook D., Moulton E.A., Tully S., Schmahmann J.D., Becerra L. (2008). Human cerebellar responses to brush and heat stimuli in healthy and neuropathic pain subjects. Cerebellum.

[6] Michelle Welman F.H.S., Smit A.E., Jongen J.L.M., Tibboel D., van der Geest J.N., Holstege J.C. (2018). Pain Experience is Somatotopically Organized and Overlaps with Pain Anticipation in the Human Cerebellum. Cerebellum.

[7] Coombes S.A., Misra G. (2016). Pain and motor processing in the human cerebellum. Pain.

[8] Helmchen C., Mohr C., Erdmann C., Binkofski F., Büchel C. (2006). Neural activity related to self-versus externally generated painful stimuli reveals distinct differences in the lateral pain system in a parametric fMRI study. Hum. Brain Mapp..

[9] Hofbauer R.K., Fiset P., Plourde G., Backman S.B., Bushnell M.C. (2004). Dosedependent Effects of Propofol on the Central Processing of Thermal Pain. Anesthesiology.

[10] Helmchen C., Mohr C., Erdmann C., Binkofski F. (2004). Cerebellar neural responses related to actively and passively applied noxious thermal stimulation in human subjects: A parametric fMRI study. Neurosci. Lett..

[11] Priori A., Ciocca M., Parazzini M., Vergari M., Ferruci R. (2014). Transcranial cerebellar direct current stimulation and transcutaneous spinal cord direct current stimulation as innovative tools for neuroscientists. J. Physiol..

[12] Huang Y., Su Y., Rorden C., Dmochowski J., Datta A., Parra L.C. An automated method for high-definition transcranial direct current stimulation modelling. Proceedings of the Annual International Conference of the IEEE Engineering in Medicine and Biology Society, EMBS.

[13] Guiomar R., Catoira B., Sobral M., Castilho P., Baeken C., Ganho-Avila A. (2022). Cerebellar Transcranial Direct Current Stimulation for Schizophrenia: A Current Modelling Study. Psychiatr. Danub. Abstr..

[14] Tomlinson S.P., Davis N.J., Bracewell R.M. (2013). Brain stimulation studies of nonmotor cerebellar function: A systematic review. Neurosci. Biobehav. Rev..

[15] Dutta A., Paulus W., Nitsche M.A. (2014). Facilitating myoelectric-control with transcranial direct current stimulation: A preliminary study in healthy humans. J. Neuroeng. Rehabil..

[16] Zuchowski M.L., Timmann D., Gerwig M. (2014). Acquisition of conditioned eyeblink responses is modulated by cerebellar tDCS. Brain Stimul..

[17] Ferruci R., Brunoni A.R., Parazzini M., Vergari M., Rossi E., Fumagalli M., Mameli F., Rosa M., Giannicola G., Zago S. (2013). Modulating human procedural learning by cerebellar transcranial direct current stimulation. Cerebellum.

[18] Galea J.M., Jayaram G., Ajagbe L., Celnik P. (2009). Modulation of cerebellar excitability by polarity-specific noninvasive direct current stimulation. J. Neurosci..

[19] Grimaldi G., Manto M. (2013). Anodal transcranial direct current stimulation (tDCS) decreases the amplitudes of long-latency stretch reflexes in cerebellar ataxia. Ann. Biomed. Eng..

[20] Purves D. (2004). Neuroscience.

[21] Castillo-Saavedra L., Gebodh N., Bikson M., Diaz-Cruz C., Brandao R., Coutinho L., Fregni F. (2016). Clinically Effective Treatment of Fibromyalgia Pain with High-Definition Transcranial Direct Current Stimulation: Phase II Open-Label Dose Optimization. J. Pain.

[22] DosSantos M.F., Love T.M., Martikainen I.K., Nascimento T.D., Fregni F., Cummiford C., DaSilva A.F.M. (2012). Immediate effects of tDCS on the μ-opioid system of a chronic pain patient. Front. Psychiatry.

[23] Fregni F., Boggio P.S., Lima M.C., Ferreira M.J.L., Wagner T., Rigonatti S.P., PascualLeone A. (2006). A sham-controlled, phase II trial of transcranial direct current stimulation for the treatment of central pain in traumatic spinal cord injury. Pain.

[24] Antal A., Terney D., Kühnl S., Paulus W. (2010). Anodal Transcranial Direct Current Stimulation of the Motor Cortex Ameliorates Chronic Pain and Reduces Short Intracortical Inhibition. J. Pain Symptom Manag..

[25] Bocci T., De Carolis G., Ferrucci R., Paroli M., Priori A., Valeriani M., Sartucci F. (2019). Cerebellar transcranial direct current stimulation (ctDCS) ameliorates phantom limb pain and nonpainful phantom limb sensations. Cerebellum.

[26] Sveva V., Cruciania A., Mancuso M., Santoro F., Latorre A., Monticone M., Rocchi L. (2024). Cerebellar non-invasive brain stimulation; a frontier in chronic pain therapy. J. Pers. Med..

[27] Sanchez-Leon C.A., Sanchez-Garrido Campos G., Fernadez M., Sanchez-Lopez A., Medina J.F., Marquez-Ruiz J. (2023). Somatodendritic orientation determines tDCS-induced neuromodulation of Purkinje cell activity in awake mice. bioRxiv.

[28] Barroso J., Branco P., Apkarian A.V. (2021). Brain mechanisms of chronic pain: Critical role of translational approach. Transl. Res..

[29] Coghill R.C., Sang N.C.N., Maisog J.M., Iadorola M.J. (1999). Pain intensity processing within the human brain: A bilateral, distributed mechanism. J. Neurophysiol..

[30] Seminowicz D.A., Moayedi M. (2017). The Dorsolateral Prefrontal Cortex in Acute and Chronic Pain. J. Pain.

[31] Silva A.F., Zortea M., Carvalho S., Leite J., da Silva Torres I.L., Fregni F., Caumo W. (2017). Anodal transcranial direct current stimulation over the left dorsolateral prefrontal cortex modulates attention and pain in fibromyalgia: Randomized clinical trial. Sci. Rep..

[32] Saab C.Y., Kawasaki M., Al-Chaer E.D., Willis W.D. (2001). Cerebellar cortical stimulation increases spinal visceral nociceptive responses. J. Neurophysiol..

[33] Souza A., Martins D.F., Medeiros L.F., Nucci-Martins C., Martins T.C., Siteneski A., Caumo W., dos Santos A.R.S., Torres I.L.S. (2018). Neurobiological mechanisms of antiallodynic effect of transcranial direct current stimulation (tDCS) in a mice model of neuropathic pain. Brain Res..

[34] Saab C.Y., Wilis W.D. (2003). The cerebellum: Organization, functions and its role in nociception. Brain Res..

[35] Witney A. (2020). From Mechanisms to Analgesia: Towards the Use of Non-Invasive Neuromodulation for Pain Relief in the Clinic.

[36] Klaus J., Schutter D.J.L.G. (2021). Electrode montage-dependent intracranial variability in electric fields induced by cerebellar transcranial direct current stimulation. Sci. Rep..

[37] Batsikadze G., Rezaee Z., Chang D.I., Gerwig M., Herlitze S., Dutta A., Nitsche M.A., Timmann D. (2019). Effects of cerebellar transcranial direct current stimulation on cerebellar-brain inhibition in humans: A systematic evaluation. Brain Stimul..

[38] Ferruci R., Cortese F., Priori A. (2015). Cerebellar tDCS: How to Do It. Cerebellum.

[39] Li S., Tang Y., Zhou Y., Ni Y. (2024). Effects of Transcranial Direct Current Stimulation on Cognitive Function in Older Adults with and without Mild Cognitive Impairment: A Systematic Review and Meta-Analysis of Randomized Controlled Trials. Geriatr. Gerontol..

